# Homicide in Chile: Trends 2000 – 2012

**DOI:** 10.1186/s12888-015-0632-5

**Published:** 2015-12-15

**Authors:** Tamara Otzen, Antonio Sanhueza, Carlos Manterola, Tamara Melnik, Monica Hetz

**Affiliations:** Doctorado en Ciencias Médicas, Universidad de La Frontera, Avenida Alemania 0458, Temuco, Chile; Escuela de Psicología, Universidad Autónoma de Chile, Temuco, Chile; Universidad Científica del Sur, Lima, Peru; Programa de Pós-graduação em Saúde Baseada em Evidências, Universidade Federal de São Paulo, São Paulo, Brazil; Departamento de Matemática y Estadísticas, Universidad de La Frontera, Temuco, Chile; Pan American Health Organization/Regional Office of the World Health Organization, Washington, USA; Departamento de Cirugía, Universidad de La Frontera, Temuco, Chile; Psychology, Catholic University of Temuco, Temuco, Chile

**Keywords:** Homicide, Aggression, Mortality, Cause of Death, Chile, South America, Latin America, Assault

## Abstract

**Background:**

Homicide, an external cause of morbidity and mortality, caused 473,000 deaths worldwide in 2012, a rate of 6.2 per 100,000 inhabitants. The aim of this study was to describe homicide mortality trends in Chile between 2000 and 2012 by year, gender, age group, geographic distribution (by zone and by region) and type of homicide.

**Methods:**

This was a population-based study. Data for homicide mortality in Chile between 2000 and 2012 were used and they were provided by the Chilean Ministry of Health’s Department of Statistics and Health Information (DEIS) and PAHO/WHO. The homicide mortality rates were calculated per 100,000 inhabitants. The study variables were year, geographic distribution, gender, age group and type of homicide. The annual percentage change (APC) of the rates was analyzed, and a logarithm of the rates by year and region was fitted by applying linear regression models. In addition, relative risks (RR) were calculated. 95 % confidence intervals were considered in all the analyses.

**Results:**

The average yearly rate of homicide (HMR) in Chile (2000–2012) was 4.9. The rates were higher in men (8.7) than in women (1.1), with a RR of 8.2. The rates were higher in the country’s central zone (5.0), increasing in recent years in the southern zone, with a significant positive APC of 1.1 %. The Aisén Region had the highest rate (7.6), although Antofagasta was the region with the most significant APC (3.1 %). The highest rate (9.2) was verified in the 25 to 39 age group. The highest rate (5.5) was recorded in 2005. The most frequent type of homicide was assault with an object (44.8 %).

**Conclusions:**

Although the homicide rates are higher in the southern zone of the country, the northern zone is showing a tendency to increase, becoming an even more serious problem, which not only affects those directly involved, but society as a whole.

## Background

Violence has been increasing in recent years, becoming a relevant topic in society and for public health. It has also become the first piece of information used by the international community to analyze the state of a country’s public safety, and decisions are made on this basis [1, 2].

Sometimes violence leads to death. There are several causes of violent death, including suicide, traffic accidents and homicide, which is defined as “unlawful death purposefully inflicted by a person on a person” [1, 3].

Homicide (also defined as “aggression”) is among the external causes of morbidity and mortality categorized by the International Statistical Classification of Diseases and Related Health Problems, Tenth Edition (ICD-10), between codes X85 and Y09. This is how the manner of death has been divided: drugs, medicament, biological substance, chemical agent, hanging, strangulation, suffocation, drowning, submersion, firearm discharge, explosive material, smoke, fire, flames, sharp objects, physical assault, crashing of motor vehicle, use of bodily force, neglect, abandonment, and others [1, 4].

According to calculations by the United Nations Office on Drugs and Crime (UNODC) Homicide Statistics, there were 437,000 deaths by homicide in 2012, a rate of 6.2 per 100,000 inhabitants [3]. Homicide rates vary considerably around the world, from lower than 3 to higher than 30. In Europe and Asia the rates remain constant, but in the Americas they have increased year on year, with a rate of 16.3 per 100,000 inhabitants in 2012. Over a third (36 %) of homicides worldwide occurs in the Americas. In South America, this variability is even greater, with rates ranging between 3.9 and 122.0 per 100,000 inhabitants. Chile reported a rate of 3.9 per 100,000 inhabitants in 2010 [3, 5].

In this context, statistics on causes of death are a useful tool for collecting data on the problems of a population’s safety and coexistence, as well as the orientation of public safety programs (central focus of public policies) [6].

In this light, the aim of this study was to describe the homicide mortality trends in Chile between 2000 and 2012.

## Methods

### Design

Population-based study.

### Study population

The databases used were provided by the National Office of the Chilean Forensic Medical Service, Pan-American Health Organization (PAHO), and the regional office of the World Health Organization (WHO) for the Americas.

### Study variables

Year, gender, age group, geographic distribution and type of assault according to the ICD-10 from codes X85 to Y09 [4]. The variable age was grouped into 5 categories: minors under 14; 15 to 24; 25 to 39; 40 to 59; and 60 and over. The geographic distribution was handled in two ways: each region independently and grouped into three zones: north (regions of Tarapacá, Antofagasta, Arica and Parinacota, Atacama, and Coquimbo); center (regions of Valparaíso, Libertador Bernardo O’Higgins, Maule and Metropolitan); and south (regions of Bío Bío, La Araucanía, Los Lagos, Los Ríos, Aisén, Magallanes and Chilean Antarctica).

#### Data analysis

Exploratory analyses were performed using the raw data. Descriptive statistics were applied, calculating percentages and measures of central tendency and variability. Gross mortality rates by homicide per 100,000 inhabitants were calculated and this information was used to create the tables and graphs. The average rates were calculated, considering the percentage of representation of inhabitants in the entire country according to the number of inhabitants per region. These data were collected from the Chilean National Institute of Statistics (NIS) [7, 8].

Then, analyses were made of the annual percentage change (APC) of the rates, using a linear regression models with their respective 95 % confidence intervals (CI) and *p* value [9].

Finally, relative risks (RR) were determined by gender, age group, and geographic distribution, calculating their respective APC and 95 % CI [10, 11].

The analyses were performed using the STATA v. 9.0 statistics software.

### Ethics

This study did not require the application of ethical safeguards, as official Chilean government statistics were used for the analyses.

## Results

In Chile between 2000 and 2012 there were 10,377 deaths by homicide, of which 9,221 (88.9 %) were men. The homicide mortality rate (HMR) in this period was 4.9 (Table 1). 2005 had the highest HMR (5.5) and 2010 the lowest (3.9) (Table 2).

**Table 1:** Homicide mortality rate per 100,000 population with their respective 95% CI

	Rate	APC	CI		*p*
General rate	4.86	-0.68	-1.25	-0.10	0.04
Men	8.73	-0.73	-1.35	-0.10	0.05
Women	1.07	-0.31	-0.83	0.22	0.28
North Zone	4.22	2.36	1.45	3.28	0.88
Center Zone	5.00	-0.16	-0.76	0.44	0.22
South Zone	4.84	1.05	0.30	1.76	0.04
Arica and Parinacota	4.40	-0.84	-2.65	1.01	0.39
Tarapacá	5.72	-0.11	-1.85	1.66	0.91
Antofagasta	3.99	3.07	1.52	4.64	0.00
Atacama	3.50	-4.05	-8.34	0.44	0.10
Coquimbo	4.02	-0.90	-2.49	0.72	0.30
Valparaíso	3.44	-3.26	-7.37	1.03	0.16
Metropolitan	5.37	-0.71	-1.36	-0.04	0.06
L. B. O'Higgins	4.69	-0.07	-1.64	1.52	0.93
Maule	5.41	0.76	-0.92	2.47	0.40
Bío Bío	3.51	-1.16	-3.01	0.73	0.25
La Araucanía	5.38	-2.10	-3.58	-0.59	0.02
Los Ríos	6.41	-1.06	-2.34	0.24	0.14
Los Lagos	6.48	-0.31	-1.22	0.61	0.52
Aisén	7.59	1.77	-1.47	5.12	0.31
Magallanes and Ch. Ant.	4.52	1.52	-2.70	5.93	0.50
0 to 14	0.20	0.32	-2.45	3.17	0.83
15 to 24	3.19	-1.44	-2.16	-0.71	0.00
25 to 39	9.15	-0.32	-0.85	0.21	0.27
40 to 59	5.78	-0.16	-2.01	1.74	0.87
60 or older	4.04	-1.09	-1.99	-0.18	0.04

**Table 2:** Homicide mortality rate per 100,000 population, by year and gender

Year	*n*	Rate	Men	Women
2000	722	4.69	8.39	1.07
2001	819	5.26	9.43	1.17
2002	792	5.03	9.07	1.07
2003	816	5.13	9.37	0.97
2004	818	5.08	9.19	1.06
2005	902	5.54	9.90	1.28
2006	841	5.12	9.32	1.00
2007	819	4.93	8.81	1.13
2008	804	4.80	8.50	1.17
2009	893	5.28	9.59	1.04
2010	666	3.90	6.80	1.05
2011	803	4.66	8.39	1.00
2012	682	3.92	6.96	0.94

Contrasting HMR by gender verified that the HMR were always higher in men than women. In 2005 both men and women presented the highest HMR (9.9 and 1.3, respectively); in 2010, the men had the lowest HMR (6.8), a situation that occurred with women in 2012 (0.9) (Table 2).

In relation to the HMR in the different age groups, from 2000 to 2012 the highest mortality rate was verified in the 25 to 39 age group (9.2); the lowest rate was in the under-14 group (0.2). These differences remained constant every year except in 2010, when the over-60 group had the second-lowest rate (Table 1).

**Fig. 1:**
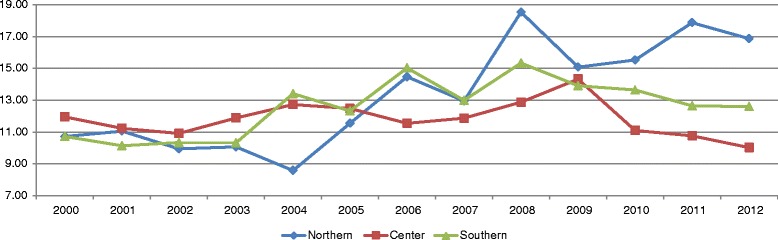
Homicide mortality rate per 100,000 population by zone

Per region the highest HMR was in Aisén (7.6) and the lowest in Valparaiso (3.4) (Table 1). When analyzing by geographic zones, it was observed that the zone with the highest HMR was the center (5.0) (Fig. 1).

Considering the ways in which homicide is committed, between 2000 and 2012 there were 3,633 homicides due to “assault by sharp object” (X99) (42.9 %); and 2,461 deaths due to “assault by firearm discharge” (X93-X95) (29.1 %). The other causes are detailed in Table 3. When the types of homicide by year, gender and age group are ungrouped, the same trends repeat.

**Table 3:** Types of homicides with their respective percentages and annual percentage change with 95% CI

	*n*	%	APC	CI		*p*
Assault by substances (X85–X90)	13	0.2%	0.99	-26.58	38.93	0.95
Assault by hanging, strangulation, suffocation, drowning and submersion (X91–X92)	250	3.0%	1.33	-0.07	2.75	0.10
Assault by firearm discharge (X93–X95)	2461	29.1%	0.13	-0.68	1.90	0.76
Assault by explosive material, smoke, fire or flames (X97–X98)	43	0.5%	4.65	-4.42	14.58	0.36
Assault by sharp object (X99)	3633	42.9%	0.40	-0.57	1.37	0.45
Assault by blunt object (X00)	162	1.9%	4.80	2.51	7.15	0.00
Assault by bodily force (Y01–Y05)	23	0.3%	-6.09	-22.86	14.32	0.55
Neglect or abandonment (Y06)	26	0.3%	-1.06	-23.91	28.64	0.94
Other maltreatment syndromes (Y07)	35	0.4%	3.64	-15.09	26.50	0.73
Assault by other specified means (Y08)	40	0.5%	7.89	-9.79	29.03	0.43
Assault by other unspecified means (Y09)	1778	21.0%	-2.71	-4.56	-0.82	0.02

In terms of the trend of types of death by homicide year on year, the most stable cause is “assault by firearm discharge” (X93-X95), with an APC of 0.13 % (CI = −0.7-1.9, *p = 0.8*). However, “assault by other specified means” (Y08) has the highest APC with 7.9 % (CI = −9.8-29.0, *p = 0.4*) (Table 3).

The homicide mortality rate between 2000 and 2012 had an APC of −0.7 (CI = −1.3- -0.1, *p = 0.07*), denoting a significant decrease in the rates of 0.7 % observed and predicted every year (Table 1).

Men presented a higher APC −0.7 % (CI = −1.4- -0.1, *p < 0.05*), with an annual decrease in their rates (Table 1).

When analyzing by age group, we observed that the highest APC was the 15 to 24 group (−1.4 % [CI = −2.2- -0.7, *p* < 0.01]) (Table 1).

The trend analysis among regions revealed that the region of Libertador Bernardo O’Higgins was the most stable (APC = −0.1 % [CI = −1.6-1.5, *p = 0.9*]) and the Atacama region had the highest APC (−4.1 % [CI = −8.3-0.4, *p = 0.10*]). The only ones with significant APC were Antofagasta and La Araucanía ((APC = 3.1 % [CI = 1.5-4.6, *p < 0.01*]) and (APC = −2.1 % [CI = −3.6- -0.6, *p < 0.05*]), respectively), the former tending to increase year on year and the latter to decrease (Table 2).

The APC was higher in the north zone (2.4 % [CI = 1.5-3.3, *p = 0.9*]), and the only one with a significant APC was the south (1.1 % [CI = 0.3-1.8, *p < 0.05*]), which shows that every year saw an increase of 1.1 % in the HMR (Table 1).

A greater risk of homicide mortality was verified in men than women (RR of 8.2), a difference that increased through the years, revealing an APC in the RR of −0.5 % (CI = −1.2-0.3, *p = 0.2*) (Table 4).

**Table 4:** Relative risk with their 95% CI and annual percentage change of relative risks with their 95% CI

	RR	CI		APC	CI		*p*
Men/women	8.17	7.66	8.82	-0.46	-1.17	0.26	0.24
Center/North	1.23	1.09	1.38	-0.65	-1.83	0.55	0.31
Aisén/Valparaíso	3.07	0.86	5.24	5.51	1.13	10.07	0.03
25 to 39 / 0 to 14	50.16	39.55	60.77	-0.64	-1.83	0.55	0.31

The risk of homicide mortality was greater in the 25 to 39 age group than the 0 to 14 group (RR of 50.2 [CI = 39.6-60.8]), with an APC of −0.6 (CI = −1.8-0.6, *p = 0.3*) (Table 4). It was also greater in the center zone than the northern zone (RR of 1.2 [CI = 1.1-1.4]), with a non-significant APC of −0.7 % (CI = 1.8-0.6, *p = 0.3*) (Table 4).

The highest risk of homicide mortality was found in the region of Aisén compared to Valparaiso (RR of 3.1 [CI = 0.9-5.2]), with an APC of 5.5 (CI = 1.1- 10.1, *p* < 0.05) (Table 4).

## Discussion

Homicide rates perhaps do not explain the entire state of a country’s security, nor perhaps do they bear a direct relation with a population’s fear of crime; their significance lies essentially in the fear of losing the main legal principle the State must protect: the human right to life [1].

Although the results of this study echo some worldwide trends in terms of HMR with respect to the variables gender and age group, important variations were found in the variables year, regions and homicide methods. We observed, for example, that the general HMR has a significant negative APC, but in the south and the region of Antofagasta the APC were positive and significant, which is why it is difficult to determine with clarity what is happening on the national level. Likewise, a year-on-year analysis of the regional rates revealed that these fluctuate in each region, possibly due to the number of homicides committed each year generally being low.

One problem in terms of how homicide is committed lies in the high percentage (21 %) of “assault by other unspecified means” (Y09), which shows that, despite the effort to provide information that enables the creation of public policies to tackle the issue, the quality of the records is very unhelpful in this regard. There are problems related to sub-records and classifications which render it difficult to ascertain the true magnitude of the problem, and they therefore limit the comparability of the data between countries [12].

The decision was made in this study not to include codes Y35-Y36, related to “legal intervention and operations of war”, which are sometimes included in analyses of homicide rates, particularly in those countries at war, a situation that does not apply to Chile [4]. “Assaults of undetermined intent” (Y10-Y34) were not analyzed either: these are normally weighed by all the external causes in order to include them in analyses [4]. It would be interesting to incorporate these codes for analysis in future studies.

In future it would also be relevant to contrast the situation in Chile with health inequality indices so as to gain a clearer picture of whether HMR are associated with population sociodemographic characteristics, and thus provide information that could enable collaboration and focus operations in the most affected sectors.

We hope that the information gained from this investigation will be of use not only to those who conduct research in this area, but also to those who have direct and indirect links to the decision-making process for public social and health policies. In this respect, it may be useful to contrast the Chilean situation with others around the world to see what countries in similar situations have done in terms of applying measures that have proven effective in these situations, and thus be able to tackle this complex situation efficiently.

Although Chile generally has low HMR, we know there are factors that could help change this situation. One has been the focus of previous studies, which determined that the countries in the Southern cone manufacture firearms [13], which in Chile represents 29.1 % of all homicide fatalities. Another factor is associated with the high immigration rates, especially in northern Chile [14].

In Chile the organization in charge of homicide analysis is the Investigative Police (PDI) [15]; such analysis could be improved with the creation of an entity in charge of quality of life, given that the topic of homicide and its collateral aspects are too complex to be approached and analyzed by a single institution.

It is hoped that this study will be of use particularly in the area of forensic psychiatry and psychology in Chile, as this is where the professionals are directly linked to homicides and their victims, considering that using knowledge of the country’s reality they can place special emphasis on the factors associated with this phenomenon in order to focus efforts on the prevention and treatment of the most affected sectors. We also hope that these results give rise to future research in the area of forensic psychiatry and psychology in Chile.

## Conclusion

This study is the first to describe homicide mortality trends in Chile. We hope that this will contribute to the situation in Chile, not only as information, but also as a tool for decision-making and implementing any policies that may help mitigate HMR.
