# Erratum to: Homicide in Chile: Trends 2000 – 2012

**DOI:** 10.1186/s12888-016-0723-y

**Published:** 2016-01-28

**Authors:** Tamara Otzen, Antonio Sanhueza, Carlos Manterola, Monica Hetz, Tamara Melnik

**Affiliations:** Doctorado en Ciencias Médicas, Universidad de La Frontera, Avenida Alemania 0458, Temuco, Chile; Escuela de Psicología, Universidad Autónoma de Chile, Temuco, Chile; Universidad Científica del Sur, Lima, Peru; Programa de Pós-graduação em Saúde Baseada em Evidências, Universidade Federal de São Paulo, São Paulo, Brazil; Departamento de Matemática y Estadísticas, Universidad de La Frontera, Temuco, Chile; Pan American Health Organization/Regional Office of the World Health Organization, Washington, USA; Departamento de Cirugía, Universidad de La Frontera, Temuco, Chile; Psychology, Catholic University of Temuco, Temuco, Chile

After publication of this work [1], we noted that the given order of the authors was incorrect. The list of authors has now been corrected in the original article, and both the corrected and original lists are reported below. We apologize for any inconvenience this oversight may have caused.

Original author list:

Tamara Otzen^1,2,3,4*^, Antonio Sanhueza^5,6^, Carlos Manterola^1,7^, Tamara Melnik^4^ and Monica Hetz^8^

Corrected author list:

Tamara Otzen^1,2,3,4*^, Antonio Sanhueza^5,6^, Carlos Manterola^1,7^, Monica Hetz^8^ and Tamara Melnik^4^
